# Supplementary material for: Highly Enhanced Photocatalytic Performances of Composites Consisting of Silver Phosphate and N-Doped Carbon Nanomesh for Oxytetracycline Degradation
Source: Int J Environ Res Public Health. 2022 Nov 11;19(22):14865. doi: 10.3390/ijerph192214865 (PMC9690370; doi:10.3390/ijerph192214865)
Supplement: Supplementary file 1 [file ijerph-19-14865-s001.zip › ijerph-2017501-supplementary.pdf]

Supporting Information

Highly Enhanced Photocatalytic Performances of Composites Consisting of Silver Phosphate and N-Doped Carbon Nanomesh for Oxytetracycline Degradation

Shehua Tong [1,2], Zhibing Liu [1], Yan Lin [1] and Chunping Yang [1,2,3,*]

1 College of Environmental Science and Engineering, Hunan University and Key Laboratory of Environmental Biology and Pollution Control (Hunan University), Ministry of Education, Changsha 410082, China

2 Guangdong Provincial Key Laboratory of Petrochemical Pollution Processes and Control, Key Laboratory of Petrochemical Pollution Control of Guangdong Higher Education Institutes, School of Environmental Science and Engineering, Guangdong University of Petrochemical Technology, Maoming 525000, China

3 School of Environmental and Chemical Engineering, Nanchang Hangkong University, Nanchang 330063, China

* Correspondence: yangc@hnu.edu.cn

2. Materials and Methods

2.2. Preparation of Ag_3PO_4

In the preparation process , $Na_2HPO_4·12H_2O$ aqueous solution (25 mL, 3 mmol) was added dropwise to $AgNO_3$ aqueous solution (25 mL, 9 mmol), and magnetic stirring was performed in the dark for 6 h. The golden yellow sediment was collected, washed with deionized water several times, and finally dried in vacuum (55°C) overnight [1].

2.4. Synthesis of Ag_3PO_4@NDC

First, 1.0 g NDC was placed in a flask containing 350 mL HNO_3 and stirred magnetically at 120 ℃ and refluxed for 8 h. Nitrogen doped carbon defect (NDC) carbon nanonets were separated by filtration, washed with deionized water until the pH value is 7, and finally dried overnight at 60 ℃. Secondly, a certain amount of NDC was dispersed into deionized water for 3 h by ultrasonic treatment. Finally, add 25 mL, 9 mmol $AgNO_3$ aqueous solution to the above NDC suspension, and then stir vigorously in the dark for 12 h. Then add $Na_2HPO_4·12H_2O$ aqueous solution (25 mL, 3 mmol) drop by drop to the mixture, and keep stirring it under dark conditions for 6 h. Finally, wash the sediment with deionized water several times, and dry it in vacuum (55 ℃) overnight [2].

2.5. Characterization

2.6. Photocatalysis Experiment

When testing oxytetracycline，the mobile phase of OTC was methanol-water (volume ratio: 60/40), the flow rate was 1 mL/min, the injection volume is 20 uL, and the column temperature was 30 ℃ [3].

3. Results and Discussion

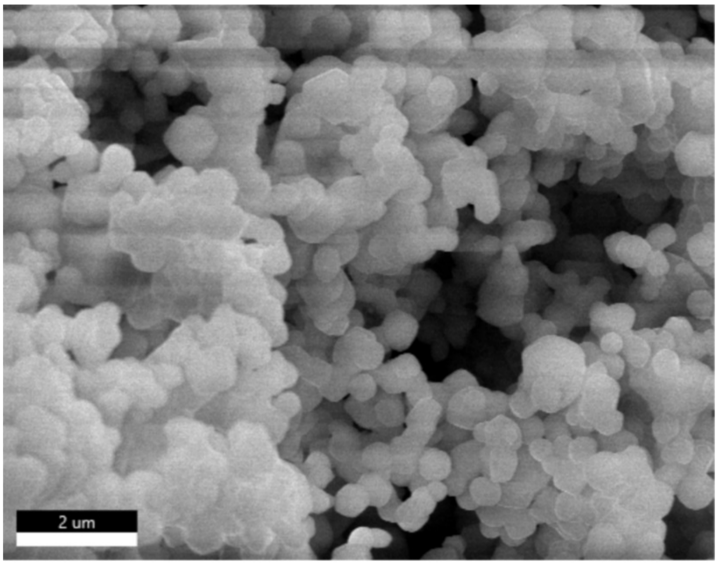

Figure S1. The origin SEM image of element distribution of Ag_3PO_4@NDC composite.

Table S1. Comparison of catalytic ability of Ag_3PO_4@NDC with other reported materials towards OTC removal.

Photocatalysts	Pollutant Concentration (mg/L)	Catalysts Dosage (g/L)	Time (min)	Removal (%)	Rate Constants (min^{-1})	References
Ag_3PO_4@NDC	20	0.5	3	100	1.6	this study
CoFe@NSC	50	0.2	150	82.7%	0.00765	[4]
Nb_2O_5/g-C_3N_4	20	1.0	150	76.2%	0.01	[5]
BPTCN	10	1.0	60	81.05%	0.0276	[6]
OCN-24-550	20	1.0	120	85.76%	0.0164	[7]
Cu_3P-$ZnSnO_3$-g-C_3N_4	10	1.0	60	54.71%	-	[8]
FeOOH QDs/CQDs/g-C_3N_4	10	0.5	60	85.5%	0.0333	[9]

References

[1] Lin, Y.; Wu, S.; Li, X.; Wu, X.; Yang, C.; Zeng, G.; Peng, Y.; Zhou, Q.; Lu, L. Microstructure and performance of Z-scheme photocatalyst of silver phosphate modified by MWCNTs and Cr-doped $SrTiO_3$ for malachite green degradation. Applied Catalysis B: Environmental 2018, 227, 557-570, doi: 10.1016/j.apcatb.2018.01.054.

[2] Yang, X.; Cui, H.; Li, Y.; Qin, J.; Zhang, R.; Tang, H. Fabrication of Ag_3PO_4-Graphene Composites with Highly Efficient and Stable Visible Light Photocatalytic Performance. ACS Catalysis 2013, 3, 363-369, doi:10.1021/cs3008126.

[3] Lin, Y.; Wu, S.; Yang, C.; Chen, M.; Li, X. Preparation of Size-Controlled Silver Phosphate Catalysts

and Their Enhanced Photocatalysis Performance via Synergetic Effect with MWCNTs and PANI. Applied Catalysis B: Environmental 2019, 245, 71-86, doi:10.1016/j.apcatb.2018.12.048.

[4] Zhang, S.; Zhao, S.; Huang, S.; Hu, B.; Wang, M.; Zhang, Z.; He, L.; Du, M. Photocatalytic degradation of oxytetracycline under visible light by nanohybrids of CoFe alloy nanoparticles and nitrogen-/sulfur-codoped mesoporous carbon. Chemical Engineering Journal 2021, 420, 130516. doi: 10.1016/j.cej.2021.130516.

[5] Hong, Y.; Li, C.; Zhang, G.; Meng, Y.; Yin, B.; Zhao, Y.; Shi, W. Efficient and stable Nb_2O_5 modified g-C_3N_4 photocatalyst for removal of antibiotic pollutant. Chemical Engineering Journal 2016, 299, 74-84, doi: 10.1016/j.cej.2016.04.092.

[6] Wang, W.; Niu, Q.; Zeng, G.; Zhang, C.; Huang, D.; Shao, B.; Zhou, C.; Yang, Y.; Liu, Y.; Guo, H.; Xiong, W.; Lei, L.; Liu, S.; Yi, H.; Chen, S.; Tang, X. 1D porous tubular g-C_3N_4 capture black phosphorus quantum dots as 1D/0D metal-free photocatalysts for oxytetracycline hydrochloride degradation and hexavalent chromium reduction. Applied Catalysis B: Environmental 2020, 273, 119051, doi:10.1016/j.apcatb.2020.119051.

[7] Guo, H.; Niu, C.; Feng, C.; Liang, C.; Zhang, L.; Wen, X.-J.; Yang, Y.; Liu, H.; Li, L.; Lin, L. Steering exciton dissociation and charge migration in green synthetic oxygen-substituted ultrathin porous graphitic carbon nitride for boosted photocatalytic reactive oxygen species generation. Chemical Engineering Journal 2020, 385, 123919, doi: 10.1016/j.cej.2019.123919.

[8] Guo, F.; Huang, X.; Chen, Z.; Cao, L.; Cheng, X.; Chen, L.; Shi, W. Construction of Cu_3P-$ZnSnO_3$-g-C_3N_4 p-n-n heterojunction with multiple built-in electric fields for effectively boosting visible-light photocatalytic degradation of broad-spectrum antibiotics. Separation and Purification Technology 2021, 265, 118477, doi: 10.1016/j.seppur.2021.118477.

[9] Zhang, M.; Lai, C.; Li, B.; Xu, F.; Huang, D.; Liu, S.; Qin, L.; Fu, Y.; Liu, X.; Yi, H.; Zhang, Y.; He, J.; Chen, L. Unravelling the role of dual quantum dots cocatalyst in 0D/2D heterojunction photocatalyst for promoting photocatalytic organic pollutant degradation. Chemical Engineering Journal 2020, 396, 125343, doi: 10.1016/j.cej.2020.125343.
